# Diversity and Distribution of *Phytophthora* Species Along an Elevation Gradient in Natural and Semi-Natural Forest Ecosystems in Portugal

**DOI:** 10.3390/pathogens14010103

**Published:** 2025-01-20

**Authors:** Carlo Bregant, Eduardo Batista, Sandra Hilário, Benedetto Teodoro Linaldeddu, Artur Alves

**Affiliations:** 1 Dipartimento Territorio e Sistemi Agro-Forestali, Università degli Studi di Padova, Viale dell’Università, 16, 35020 Legnaro, Italy; benedetto.linaldeddu@unipd.it; 2 Centre for Environmental and Marine Studies (CESAM), Departamento de Biologia, Universidade de Aveiro, 3810-193 Aveiro, Portugal; eduardobatista@ua.pt (E.B.); hilario.sandra@fc.up.pt (S.H.); artur.alves@ua.pt (A.A.); 3 GreenUPorto—Sustainable Agrifood Production Research Centre/Inov4Agro, Department of Geosciences, Environment and Spatial Plannings (DGAOT), Faculty of Sciences, University of Porto, Campus de Vairão, Rua da Agrária 747, 4485-646 Vila do Conde, Portugal

**Keywords:** emerging diseases, invasive pathogens, diversity, host jump, oomycetes

## Abstract

Globally, forests are constantly threatened by a plethora of disturbances of natural and anthropogenic origin, such as climate change, forest fires, urbanization, and pollution. Besides the most common stressors, during the last few years, Portuguese forests have been impacted by severe decline phenomena caused by invasive pathogens, many of which belong to the genus *Phytophthora*. The genus *Phytophthora* includes a large number of species that are invading forest ecosystems worldwide, chiefly as a consequence of global trade and human activities. This paper reports the results of a survey of *Phytophthora* diversity in natural and semi-natural forest ecosystems in Portugal along an elevation gradient. Isolations performed from 138 symptomatic plant tissues and rhizosphere samples collected from 26 plant species yielded a total of 19 *Phytophthora* species belonging to 6 phylogenetic clades, including *P. cinnamomi* (36 isolates), *P. multivora* (20), *P. plurivora* (9), *P. cactorum* (8), *P. lacustris* (8), *P. pseudocryptogea* (8), *P. amnicola* (6), *P. hedraiandra* (6), *P. pseudosyringae* (5), *P. thermophila* (5), *P. bilorbang* (4), *P. inundata* (4), *P. asparagi* (3), *P. citricola* (3), *P. gonapodyides* (3), *P. rosacearum* (3), *P. chlamydospora* (2), *P. pachypleura* (2), and *P. syringae* (1). Overall, the data obtained highlight the widespread occurrence of *P. cinnamomi* in natural ecosystems from sea level to mountain habitats. The results of the pathogenicity tests carried out on 2-year-old chestnut plants confirmed the key role of *P. cinnamomi* in the recrudescence of chestnut ink disease and the additional risk posed by *P. pachypleura*, *P. plurivora*, and *P. multivora* to Portuguese chestnut forests. Finally, three species, *P. citricola*, *P. hedraiandra*, and *P. pachypleura*, are reported for the first time in the natural ecosystems of Portugal.

## 1. Introduction

In Portugal, forests provide a variety of resources and play a primary role in the national economy [1]. About 36% of the Portuguese mainland is covered by forested areas [2]. According to the last National Forest Inventory, Portuguese forests are mainly composed of *Eucalyptus* spp. (mostly *Eucalyptus globulus*) plantations with a surface of over 845,000 hectares. The other key forest species are *Quercus suber* (720,000 ha) and *Pinus pinaster* (713,000 ha) [2].

Forest productions, such as timber, cork, pulp, paper, and wooden furniture, are an important source of income and represent approximately 2% of the national gross domestic product and 10% of total Portuguese exports [1].

In Portugal, during the past decades, a change in land management has occurred following the progressive abandonment of agricultural activities and the consequent transfer of land use towards forestry [3]. Both natural and artificial forests in Portugal are constantly threatened by disturbances of natural and anthropogenic origin. Forest fires and drought induced by climate change are currently the most serious abiotic threats to Portuguese forests, inducing phenomena of land degradation and desertification [4].

Although forest fires are natural occurrences in Mediterranean regions, the past few decades have seen a clear increase in the frequency of major forest fire events, especially in Portugal [4,5,6]. Portugal is the European country with the highest percentage of its forestry land lost to wildfires between 2001 and 2021, about 13% [7].

Climate influences the structure and function of forest ecosystems and plays a primary role in forest health. Rapid variation in climatic conditions can directly and indirectly affect the growth and productivity of forests through changes in temperature, humidity, precipitation, and other factors [8,9]. In the last three decades, a significant reduction in summer rainfall characterized the Iberian Peninsula, alternating with extreme events of concentrated rain [10,11]. Severe summer drought events combined with the spread of fire-prone forest species such as *E. globulus* and *P. pinaster* create the most suitable conditions for the occurrence of large-scale fires on the Portuguese mainland [12].

Abiotic disturbances and stress conditions can intensify many of the biotic threats to forests, such as the outbreak of invasive pests and pathogens [9,13]. In recent years, a drastic decline in forest ecosystems has characterized large areas of Portugal [14,15,16]. The low diversity of forest species and the dominant occurrence of clonal stands could favour the occurrence of large outbreaks of forest pests and pathogens, at either spatial or temporal scales, whenever disturbances occur [14]. Pine and cork oak forests are the formations most affected by multiple attacks of insects and fungi [14].

Among the most serious pathogens threatening European forests, some species belonging to the family *Botryosphaeriaceae* (Ascomycota) and the genus *Phytophthora* (Oomycota) have been assuming a primary role in recent decades [17,18]. These invasive and often polyphagous pathogens can simultaneously affect plants, causing serious decline phenomena [19,20,21].

In Portugal, some recent studies have identified the widespread occurrence, in forest ecosystems, of 22 species of *Botryosphaeriaceae* [15,22,23,24,25], whereas, until some years ago, little was known about the occurrence and diversity of oomycetes in natural ecosystems in Portugal [26,27]. Although serious outbreaks of ink disease have impacted chestnut forests since the first half of the 19th century and cork oak stands since 1900, scientific interest in *Phytophthora* disease has grown considerably only in recent years [27,28,29,30]. Recent studies have revealed a widespread presence of *P. cinnamomi* in chestnut, eucalyptus and cork oak forests, especially in the central and southern areas of the country [29,31,32,33,34,35,36]. In addition, severe *Phytophthora* outbreaks are devastating riparian and wet habitats populated by *Alnus glutinosa* in Central Portugal [27,37]. A recent study listed over 30 species and hybrids in forest ecosystems and nurseries in Portugal [38].

Therefore, given the still limited information about the *Phytophthora* species involved in the severe decline phenomena affecting natural and artificial forest formations in Portugal, a study was conducted to evaluate the diversity and distribution of *Phytophthora* species in the main habitats along an elevation gradient.

## 2. Materials and Methods

### 2.1. Field Surveys and Sampling Procedure

From February to June 2022, a preliminary survey was conducted across Portugal to evaluate the health status of forest formations. Afterwards, twenty-one different sites were randomly selected along the entire elevation gradient from the sea level (0 m a.s.l) to the highest mountains (1900 m a.s.l.). The 21 sites are representative of three different macroclimatic areas, a Mediterranean/coastal zone (A), a temperate zone (B), and a submontane/montane belt (C), according to the climatic zonation of Köppen [39].

At each site, the plants were checked for the presence of typical *Phytophthora* disease symptoms on the canopy (leaf necrosis, bleeding cankers, epicormic shoots, sudden death, and chlorosis) and root system (exudates, necrosis, and loss of fine roots). At some sites (1, 5, 6, 8, 9, 10, 11, 12, 13, 16, 17, and 21), a linear transect of 50 m was randomly established to evaluate the disease incidence and mortality rate, as reported in Bregant et al. [27] (Table 1).

A total of 138 samples were randomly collected from 26 plant host species, including rhizosphere (106 samples), necrotic leaves, and bark tissues from bleeding cankers (32) (Table 1). The plants sampled were divided into natural, planted, and invasive.

### 2.2. Isolation of Pathogens

In the laboratory, *Phytophthora* isolation was attempted as reported in Bregant et al. [27]. Rhizosphere soil samples were placed in plastic cylinders and flooded with distilled water. Young *Q. suber*, *Hedera helix*, and *Pittosporum* sp. leaves were used as bait on the water surface. Cylinders were kept at 20 °C under natural daylight and checked after 12–24 h for 3–5 days. Leaves showing dark spots were divided into small fragments of 5 mm^2^ and placed on 90 mm Petri dishes containing the selective medium PDA+ reported in Bregant et al. [40].

The isolation of *Phytophthora* species was also directly attempted from necrotic leaves and bark tissues collected from bleeding cankers. Small fragments taken with a sterile scalpel along the border of the necrosis were placed in Petri dishes containing PDA+.

The plates were incubated in the dark at 20 °C and examined every 12 h. Hyphal tips typical of *Phytophthora* from the emerging colonies were sub-cultured on potato dextrose agar (PDA) and carrot agar (CA) and incubated at 20 °C in the dark [41].

### 2.3. Identification of Pathogens

All isolates were initially divided into morphotypes based on colony growth characteristics, including their colony appearance after 7 days of incubation on PDA and CA at 20 °C in darkness, as well as the morpho-biometric data of sporangia and oogonia. All isolates were initially divided into morphotypes. *Phytophthora* isolates were grouped according to morphological descriptions provided by Erwin and Ribeiro [41]. To enhance sporangia production, CA plugs (5 mm diameter) of each isolate were placed in Petri dishes containing 10 mL of unsterile pond water with 2 mL of carrot broth added. Sporangial production was assessed every 6 h for 4 days by microscopic observation. For all isolates, breeding systems were evaluated on CA Petri dishes after 20 days of incubation at 20 °C. The biometric data of morphological structures were measured with the software Motic Images Plus 3.0 paired with a Moticam 10+ camera connected to a Motic BA410E microscope (MOTIC INSTRUMENTS INC. Viking Way, Richmond, BC, Canada).

Molecular analysis was used to confirm the identity of all isolates at the species level. The genomic DNA was extracted from the mycelium of 5-day-old cultures grown on PDA at 20 °C, according to the protocol reported by Möller et al. [42]. The primers ITS5 and ITS4 were used to amplify and sequence the internal transcribed spacer (ITS) regions, including the complete 5.8S gene [43]. Polymerase chain reaction (PCR) mixtures and amplification conditions were as described by Bregant et al. [27]. PCR amplicons were purified with the DNA NZY Gelpure kit MB01102 (Nzytech, Lisbon, Portugal) following the manufacturer’s instructions. The ITS regions were sequenced by the GATC Biotech (Cologne, Germany). The nucleotide sequences were read and edited with FinchTV 1.4.0 (Geospiza, Inc., http://www.geospiza.com/finchtv, accessed on 1 December 2024) and then compared with reference sequences (ex-type material) retrieved from GenBank using the BLASTn algorithm. ITS sequences from representative isolates obtained in this study were deposited in GenBank www.ncbi.nlm.nih.gov/genbank (accessed on 1 December 2024) (Table 2).

### 2.4. Phylogenetic Analysis

Molecular phylogeny based on ITS sequences was used to reconstruct evolutionary relationships among the *Phytophthora* species obtained in this study into the known clades of the genus [44]. Nineteen ITS sequences representative of the *Phytophthora* species obtained were compiled in a dataset together with thirty-two sequences from ex-type material of *Phytophthora* species representative of all phylogenetical clades (Table 2). Two isolates of *Halophytophthora avicenniae* and two of *Nothophytophthora caduca*, including those obtained in this study, were included as outgroup taxa.

Sequences were aligned with ClustalX v. 1.83 [45] using the parameters reported by Bregant et al. [40].

Phylogenetic reconstructions were performed with MEGA-X 10.1.8, including all gaps in the analyses. The best model of DNA sequence evolution was determined automatically by the software [46]. Maximum likelihood (ML) analysis was performed with a neighbour-joining (NJ) starting tree generated by the software. A bootstrap analysis (1000 replicates) was used to estimate the robustness of nodes.

### 2.5. Pathogenicity Test

To fulfil Koch’s postulates, the pathogenicity of six representative isolates of *Phytophthora* obtained from chestnut (*P. cactorum* CBP168, *P. cinnamomi* CBP185, *P. multivora* CBP154, *P. pachypleura* CBP158, *P. plurivora* CBP164, and *P. pseudocryptogea* CBP166) was tested against 2-year-old chestnut plants cultivated in plastic pots (1 L volume). The experimental design consisted of eight seedlings inoculated per isolate. A 5 mm diameter hole was made through the bark of the stem using a cork borer and replaced with an agar plug of the same size taken from the margin of 5-day-old cultures grown on PDA. The inoculation wounds were wrapped with sterile damp cotton wool and covered with aluminium foil. Eight seedlings were inoculated with a sterile plug of PDA as a control. Plants were kept in field conditions ranging from 9 to 29 °C and watered regularly for 30 days.

At the end of the experimental period, symptoms were checked and the extent of the external lesions was measured. Pathogenicity assay data were first checked for normality (Anderson–Darling test) and then subjected to analysis of variance (ANOVA). Significant differences among mean values were determined using Fisher’s least significant differences multiple range test (*p* = 0.05) after one-way ANOVA using XLSTAT 2008 software (Addinsoft).

Re-isolation was made from small pieces of wood removed from lesion margins onto PDA+. Growing colonies were sub-cultured onto CA and PDA, incubated in the dark at 20 °C, and identified through morphology and ITS sequencing.

### 2.6. Geographic Distribution of Phytophthora Species

A literature review was conducted, focusing on the terms “*Phytophthora*” and “Portugal” (source: Scopus, Google Scholar, and GenBank, November 2024). All relevant records containing geographic information were standardized and organized in a single dataset. Records without a clear geographical identification were not included in this analysis.

## 3. Results

### 3.1. Field Survey

Symptoms of decline and mortality were recovered in almost all monitored sites in Portugal. More specifically, emerging diseases are affecting all climatic regions in the country, ranging from the Mediterranean vegetation of the Algarve to the montane habitats in Serra da Estrela (>1900 metres a.s.l.).

**Figure 1:**
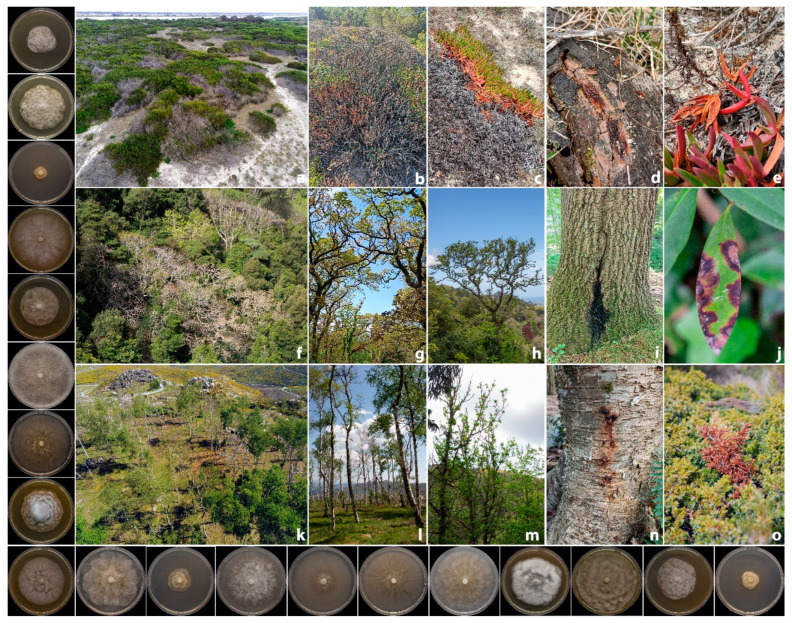
Overview of *Phytophthora* disease symptoms observed in coastal ecosystems (**a**–**e**), temperate (**f**–**j**), and montane forests (**k**–**o**) across Portugal: *Acacia longifolia* (**a**,**d**), *Pistacia lentiscus* (**b**), *Carpobrotus edulis* (**c**,**e**), *Quercus* spp. (**f**–**i**), *Rhododendron ponticum* (**j**), *Betula celtiberica* (**k**,**l**,**n**), *Castanea sativa* (**m**), and *Juniperus communis* (**o**). On the left, starting from the top, colony morphology of *Phytophthora amnicola*, *P. asparagi*, *P. bilorbang*, *P. cactorum*, *P. chlamydospora*, *P. cinnamomi*, *P. citricola*, *P. gonapodyides*, *P. hedraiandra*, *P. inundata*, *P. lacustris*, *P. multivora*, *P. pachypleura*, *P. plurivora*, *P. pseudocryptogea*, *P. pseudosyringae*, *P. rosacearum*, *P. syringae*, and *P. thermophila* after 7 days of growth at 20 °C on CA in the dark.

Affected plants showed mainly typical root and collar rot symptoms, exudates at the lower part of the stem, stunted growth, and, in severe cases, sudden death (Figure 1). In some sites, aerial *Phytophthora* symptoms were observed on different plant species, involving various plant organs such as leaves and twigs (Figure 1). Moreover, in stems and branches, extensive bleeding cankers were observed and necrosis progressively girdled the circumference of the branch, causing partial or total death of the crown (Figure 1).

Disease incidence ranged from 30 to 100% with an average mortality rate of 11–55% (Table 3). The most affected formation appeared to be the coastal maquis of *Pistacia lentiscus* and the mountain forests of *Betula celtiberica*, with a mortality rate of 31 and 55%, respectively.

### 3.2. Phytophthora Diversity in Portugal

From the 138 samples collected in different habitats across Portugal, 136 *Phytophthora* isolates were obtained belonging to six different ITS clades (Table 4). Of these, 22 isolates emerged from bleeding cankers and necrotic leaves and 114 from rhizosphere soil samples.

On the bases of morphology, colony appearance, and ITS sequence data, *Phytophthora* isolates were identified as *P. cinnamomi* (36 isolates), *P. multivora* (20), *P. plurivora* (9), *P. cactorum* (8), *P. lacustris* (8), *P. pseudocryptogea* (8), *P. amnicola* (6), *P. hedraiandra* (6), *P. pseudosyringae* (5), *P. thermophila* (5), *P. bilorbang* (4), *P. inundata* (4), *P. asparagi* (3), *P. citricola* (3), *P. gonapodyides* (3), *P. rosacearum* (3), *P. chlamydospora* (2), *P. pachypleura* (2), and *P. syringae* (1). In addition to *Phytophthora* species, two isolates of *Halophytophthora avicenniae* and two of *Nothophytophthora caduca* were obtained (Table 4).

The most common and widespread *Phytophthora* species detected in this study across Portugal was *P. cinnamomi*. This species was isolated from 12 out of the 26 hosts, in 12 sites distributed across all climatic regions. The other dominant species were *P. multivora* and *P. plurivora*, isolated from five and six hosts and four and five sites, respectively.

### 3.3. Phytophthora Distribution in Portugal

**Figure 2:**
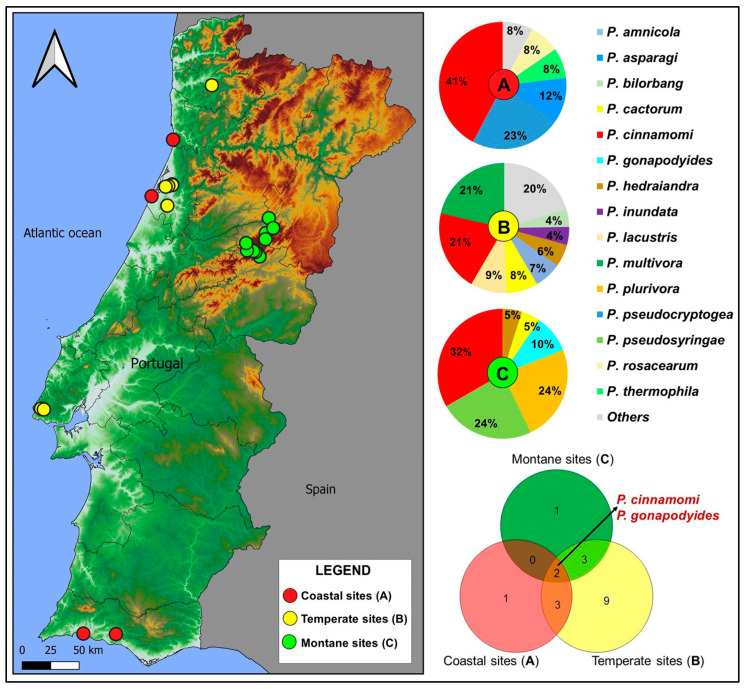
Isolation frequency and distribution of the most common *Phytophthora* species isolated in this study.

As regards the geographical distribution of the species isolated in this study, a great variability emerged according to the climatic areas. All three zones (coastal/Mediterranean, temperate, and montane regions) showed a wide diversity in *Phytophthora* assemblages (Figure 2).

Many species are typical of one or two geographical areas; only *P. cinnamomi* and *P. gonapodyides* have been isolated in all three areas spanning from sea level to the mountain belt (Figure 2). In addition to *P. cinnamomi*, *P. pseudocryptogea*, and *P. multivora* are the other most frequent species in coastal (23%) and temperate areas (21%), respectively, whereas at higher altitudes, *P. pseudosyringae* and *P. plurivora* have been isolated from 24% of the examined samples.

Combining data from the literature review with our present study, a total of 34 different known *Phytophthora* species and 3 hybrids have been isolated and officially reported in natural and semi-natural ecosystems in Portugal, including 122 host–pathogen interactions (Table 5).

**Figure 3:**
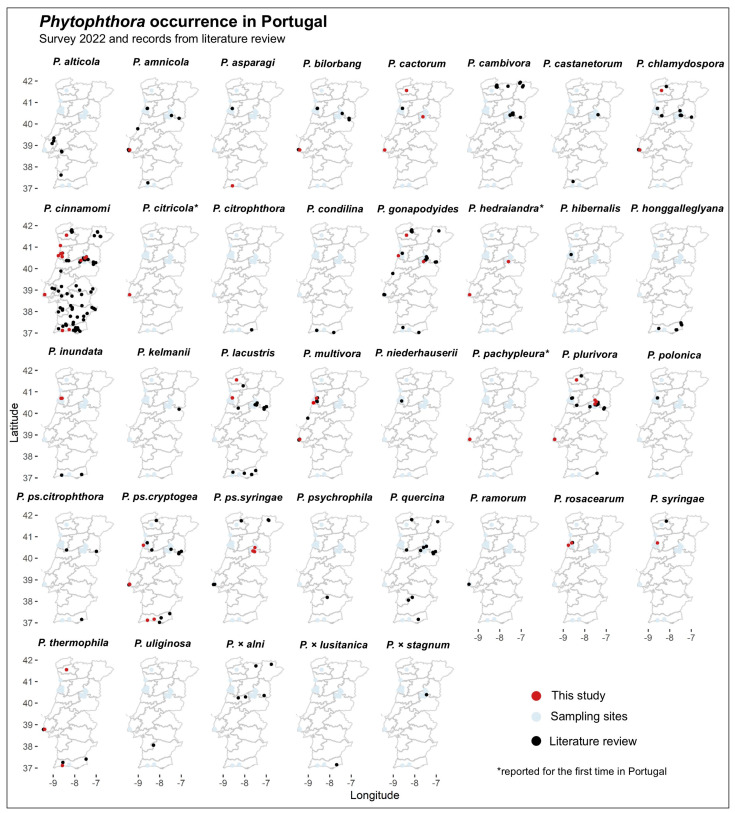
Distribution of *Phytophthora* species in Portugal. Red dots are occurrences for this study, black dots are from literature data, and blue dots in the background are for sampling areas.

**Figure 4:**
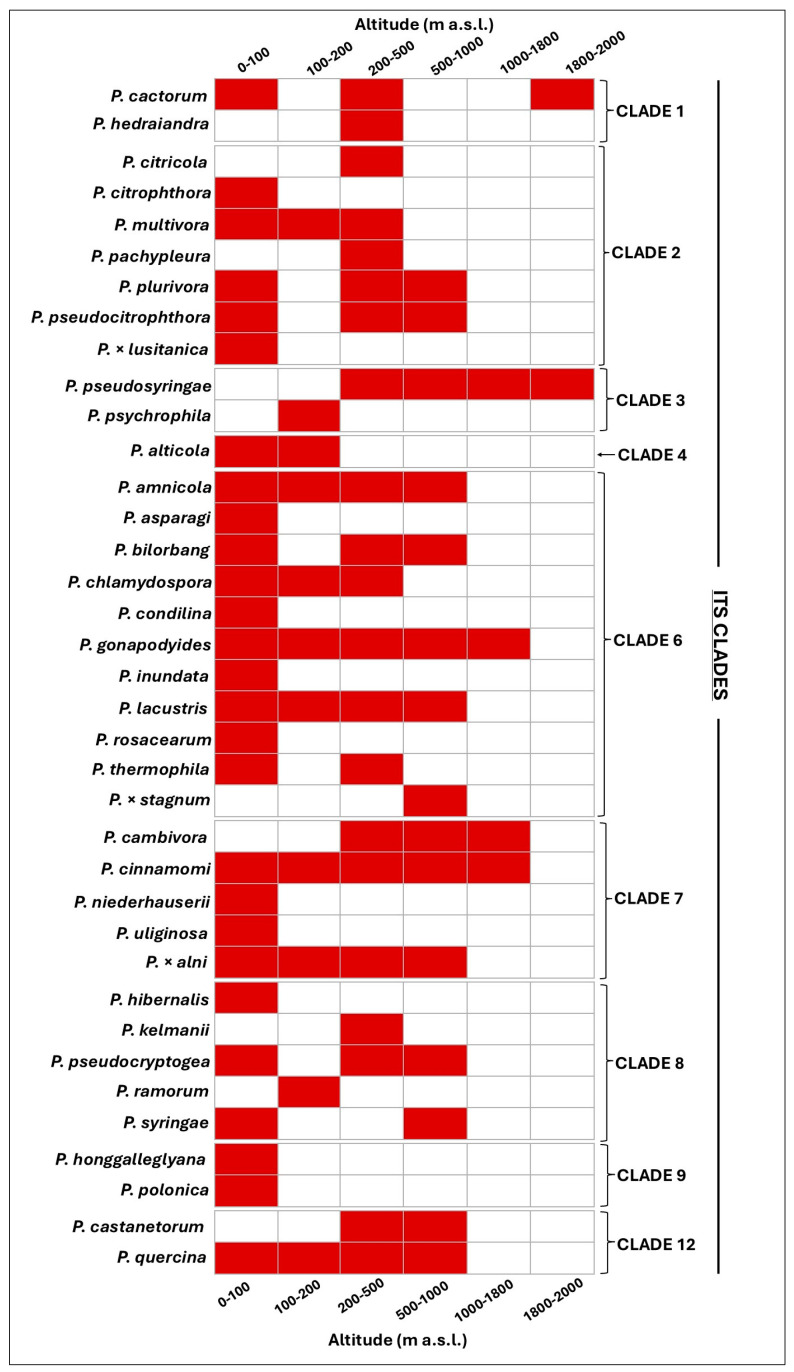
*Phytophthora* diversity along the elevation gradient in Portugal. Data from the study and literature review.

For the 37 *Phytophthora* species and hybrids for which data on isolation points are available, geographical and altitudinal distribution in Portugal’s mainland were reconstructed (Figure 3 and Figure 4).

Among the different species, *P. cinnamomi* is the most widespread from north to south and from west to east and has been reported from 29 different hosts (Table 5, Figure 3).

Other species widespread in the country are *P. gonapodyides*, *P. plurivora*, *P. pseudocryptogea*, and *P. quercina* (Figure 3). The distribution of the remaining species is more localized and fragmented (Figure 3).

Historically, the occurrence of *P. cinnamomi* appears related chiefly to *Q. suber* stands in the southern part of Portugal, but our study also revealed a common presence of this species in northern and central Portugal, including mountain areas.

The distribution along altitude shows the potential adaptability of the 37 *Phytophthora* species and hybrids to different climatic conditions (Figure 4). Most of the species reported in Portugal have been isolated at low altitudes from 0 to 500 metres a.s.l. Some species, such as *P. cactorum*, *P. cinnamomi*, and *P. gonapodyides*, manifest plasticity to all altitudes from sea level to over 1000 m a.s.l. Finally, *P. pseudosyringae* is the only species isolated exclusively in mountain forests.

### 3.4. ITS Phylogeny

**Figure 5:**
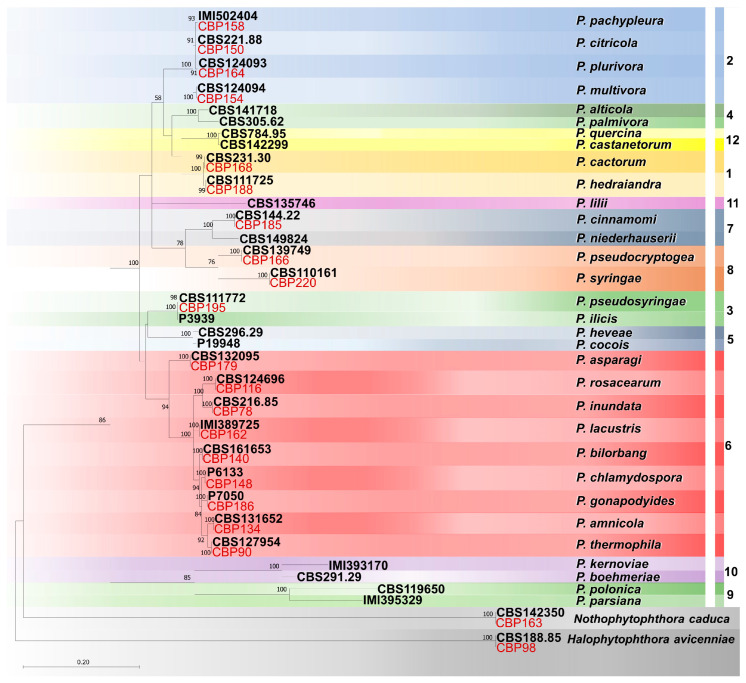
Maximum likelihood tree obtained from the internal transcribed spacer (ITS) sequences of *Phytophthora* species representative of the 12 clades. The tree was rooted to *Halophytophthora avicenniae* and *Nothophytophthora caduca*. Data are based on the General Time Reversible model. A discrete Gamma distribution was used to model evolutionary rate differences among sites. The tree is drawn to scale, with branch lengths measured in the number of substitutions per site. Bootstrap support values in percentage (1000 replicates) are given at the nodes. Ex-type cultures are in bold, and isolates obtained in this study are in red.

The phylogenetic relationships among the *Phytophthora* isolates obtained in this study were elucidated using ITS sequences (Figure 5). In particular, the isolates included in the phylogenetic analysis were distributed in 19 terminal clades with the relative ex-type of 19 formally described species (Figure 5). Isolates of *Halophytophthora avicenniae* and *Nothophytophthora caduca* obtained in this study clustered in two basal clades with the relative ex-type strains.

The 19 *Phytophthora* species belong to 6 of the 12 phylogenetic clades of this genus [44]. Among all, nine species (*P. amnicola*, *P. asparagi*, *P. bilorbang*, *P. chlamydospora*, *P. gonapodyides*, *P. inundata*, *P. lacustris*, *P. rosacearum*, and *P. thermophila*) belong to clade 6, whereas four species reside in the ex-type *P. citricola* complex in clade 2 (*P. citricola*, *P. multivora*, *P. pachypleura*, *P. plurivora*). The other clades (1, 3, 7, and 8) are represented by only one or two species.

### 3.5. Pathogenicity Test

**Figure 6:**
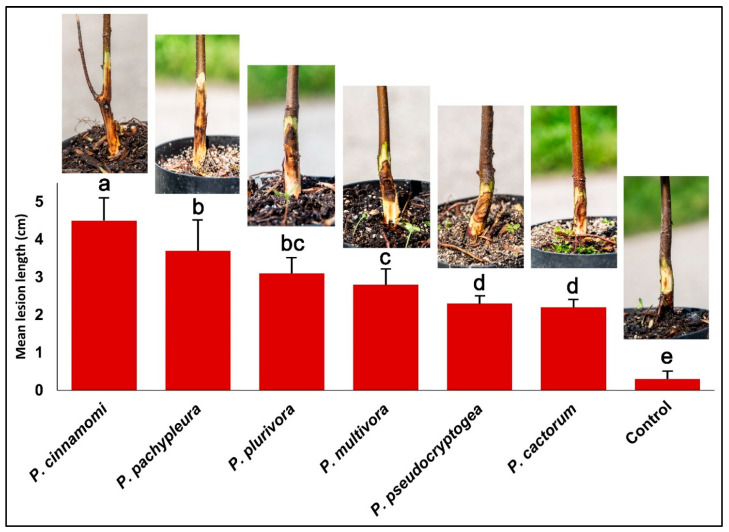
Mean lesion length (±standard deviation) and symptoms on 2-year-old seedlings of *Castanea sativa* detected after 1 month from the inoculation with *Phytophthora* spp. Values with the same letter do not differ significantly at *p* = 0.05, according to the LSD multiple range test.

All *Phytophthora* species proved to be pathogenic to chestnut plants. At the end of the experimental period, inoculated seedlings showed dark brown inner bark lesions that spread up and down from the inoculation point at the collar root (Figure 6). Among the different species assayed, the length of the necrotic lesion differed significantly (Figure 6). The lesions caused by *P. cinnamomi* were significantly larger than those caused by other species (Figure 6). Also, *P. pachypleura*, *P. plurivora*, and *P. multivora* caused large lesions, while the other species only caused small necrotic lesions. Lesions caused by *P. cinnamomi*, *P. pachypleura*, and *P. plurivora* progressively girdled the twigs, causing shoot blight, browned foliage, and wilting symptoms. Control seedlings remained asymptomatic. Re-isolation was conducted positively for 100% of seedlings inoculated with *Phytophthora* spp.

## 4. Discussion

The results obtained in this study have allowed us to clarify both the symptomatology and aetiology related to severe decline phenomena affecting natural and planted forest ecosystems in Portugal from the sea level to the mountain belt. Over the past decades, research concerning the impact of *Phytophthora* in Portuguese forests has predominantly focused on the central and southern regions of the country, especially on chestnut and cork oak trees [31,32,33,34]. Furthermore, the recent occurrence of new outbreaks has made it possible to associate a large community of *Phytophthora* spp. with extensive decline phenomena of *Eucalyptus globulus* plantings and *Alnus glutinosa* riparian systems [27,29,30,37]. Furthermore, a study conducted from 2010 to 2015 reported a high diversity of oomycetes across Portuguese forests, rivers, and nurseries [38].

Regarding this study, the field surveys conducted in twenty-one Portuguese forest systems highlight and confirm that the severe disease outbreaks and mortality are affecting several woody plant species, from the Mediterranean to the sub-montane and montane forest formations in Portugal. The most impacted formations were the coastal Mediterranean maquis dominated by *Pistacia lentiscus* and the mountain forests of *Betula celtiberica*, but *Phytophthora*-related diseases affect a multitude of plant species including chestnut, oaks, poplars, willows, and eucalyptus.

The results showed a complex of pathogenic *Phytophthora* species associated with different symptoms, including leaf and shoot blights, bleeding cankers, and root rot on twenty-six different plant species. Overall, nineteen *Phytophthora* species belonging to six different phylogenetic clades were isolated and identified by means of morphological characters and DNA sequence data. These include *Phytophthora amnicola*, *P. asparagi*, *P. bilorbang*, *P. cactorum*, *P. chlamydospora*, *P. cinnamomi*, *P. citricola*, *P. gonapodyides*, *P. hedraiandra*, *P. inundata*, *P. lacustris*, *P. multivora*, *P. pachypleura*, *P. plurivora*, *P. pseudocryptogea*, *P. pseudosyringae*, *P. rosacearum*, *P. syringae*, and *P. thermophila.* The *Phytophthora* diversity found in Portugal includes both cosmopolitan and polyphagous pathogens and rare species known to attack a limited number of plant hosts in a few geographic areas.

The most common species isolated in this study is *P. cinnamomi*. This invasive pathogen has been isolated from 12 hosts in 12 sites across different climatic regions. This confirms previous studies on the wide occurrence of this pathogen in forest systems of Portugal [29,31,34,38]. It is considered one of the most invasive organisms worldwide [54]. In our study, *P. cinnamomi* has been isolated from declining trees from the Algarve (Mediterranean maquis) to the undisturbed cold *Betula celtiberica* forests in the Serra da Estrela (over 1200 m a.s.l.). This finding highlights the strong potential and plasticity of *P. cinnamomi* to invade, survive, and adapt to different environments, including low-temperature habitats, confirming the recent distribution patterns developed for this species and the results of other recent investigations in Europe and Australia [55,56,57,58,59].

This study has significantly expanded knowledge on the diversity and impact of pathogenic oomycetes in mountainous areas of Europe. Serra da Estrela, located in Central Portugal, is the highest mountain range and largest protected area. It is characterized by heterogeneous ecological conditions based on the various slopes and altitudes from 300 to over 1900 m. a.s.l. [60]. The involvement of both airborne and soilborne *Phytophthora* species is causing extensive mortality of many natural and planted mountain species such as birch, larch, and common juniper. In addition to *P. cinnamomi*, some species belonging to clades 1 and 3 and characterized by producing caducous sporangia emerged frequently from tissue samples of different alpine hosts. These included *P. pseudosyringae*, a typical species of mountain habitats, and the more plastic *P. cactorum* and *P. hedraiandra.* As reported in two previous studies conducted in alpine formations, low cardinal temperature for growth, the production of resistant structures, and an aerial lifestyle would seem to favour the affirmation of these species in cold environments [18,40]. Interestingly, the results of this study confirm the wide diffusion of all the species belonging to clade 1a, except *P. aleatoria*, in the mountainous areas of Europe including the wildest ones not disturbed by humans [18,40,61].

The abundant production of chlamydospores and hyphal swellings by the strains of *P. cinnamomi* and *P. pseudosyringae* obtained in Serra da Estrela could explain the adaptation and resistance of these species in a very “hostile” area, characterized by extremely cold winters and very hot and dry summers with frequent forest fires.

The other two most common species obtained in this study were *P. multivora* and *P. plurivora* (clade 2). The distribution of *P. plurivora* in Europe has been documented for a long time in several countries, with population studies indicating a European origin [62]. Its occurrence in the Iberian Peninsula was associated with many hosts in forest and nursery systems [27,38,63,64,65].

However, the introduction of the invasive *P. multivora* in Europe seems recent and it is currently spreading in Mediterranean regions, thanks to its greater adaptations to heat and dry conditions [27,61,66,67]. In Portugal, *P. multivora* was recently reported on *Acacia dealbata*, *Acer pseudoplatanus*, *Alnus glutinosa*, *Eucalyptus globulus*, *Fraxinus angustifolia*, and *Quercus rubra* [27,30,38]. The new association of this pathogen with several other native and invasive plant species suggests the good adaptation of this invasive species to Portugal’s climate and confirms the polyphagous nature of this organism reported in Australia and South Africa, posing a serious threat to European forests in the face of climate changes [67,68,69,70].

In addition to *P. multivora* and *P. plurivora*, another two clade 2 species of *P. citricola sensu latu* complex, namely *P. citricola* and *P. pachypleura*, have been isolated for the first time from natural forests of sycamore maple and sweet chestnut near Sintra (temperate sites 11 and 12), respectively. *Phytophthora citricola s.s.* is a pathogen known for a long time to cause severe diseases in wood crops, although many historical reports of this species probably belong to the cryptic species *P. multivora* and *P. plurivora* [71]. *Phytophthora pachypleura* was recently described from ornamental plants in Europe [72]. Before this study, these two species had never been recovered in Portugal and in natural areas of the continent.

Six species were isolated from *Castanea sativa* in this study. The under-bark inoculation assay confirmed the aggressiveness of *P. multivora*, *P. pachypleura*, and *P. plurivora* on *Castanea sativa*, with a statistically more extensive necrosis than that of *P. pseudocryptogea* and *P. cactorum* and slightly less than that caused by *P. cinnamomi.* These results confirm that the aetiology of ink disease is rapidly evolving in Europe and now includes *P. multivora* and *P. pachypleura*, reported here for the first time as chestnut pathogens.

A conspicuous number of species found in this study belong to the ITS clade 6 *sensu* [44]. *Phytophthora* species from clade 6 have an aquatic and saprotrophic lifestyle; however, some species can act as opportunistic or aggressive tree pathogens [61,73]. This result is due to the numerous samples collected along riparian systems or wetlands and confirms the wide diversity of clade 6 species found in previous research in water systems of Portugal and Europe [27,37,38,74].

In particular, *P. gonapodyides* is widespread in all climatic areas. The other clade 6 species (*P. asparagi*, *P. bilorbang*, *P. chlamydospora*, *P. inundata*, and *P. rosacearum*) appear rarer and geographically confined [26,27,38]. Finally, the isolation of *P. thermophila* from four hosts in north, central, and south Portugal is very important and confirms the common presence of this species in Portugal, recently reported along watercourses [38]. *Phytophthora thermophila* was previously described from *Eucalyptus* forests and river systems of Australia and South Africa [75,76]. This species has high optimum and maximum temperatures for growth and a relative capacity for interspecific hybridization [75,77,78]. Some unstable hybrids close to *P. amnicola* and *P. thermophila* have also been isolated from riparian systems in this study (Bregant and Alves, unpublished). Future studies are necessary to clarify the pathogenicity of *P. thermophila* and the role of the related hybrids in order to understand the real risk posed by this pathogen to Portuguese and European forests.

Finally, combining the results of this study with the literature review data, a total of 37 species and hybrids are now officially reported in the forest ecosystems of Portugal. The distribution of *Phytophthora* covers all climatic areas investigated along the altitudinal gradient. Some species are restricted to one altimetric range whereas others such as *P. cactorum*, *P. cinnamomi*, and *P. gonapodyides* are invasive. A survey is currently in progress to clarify the pathogenicity of the new host pathogens’ association and to understand the susceptibility of native plants to the most invasive pathogens detected in this study.

Three species, *P. citricola*, *P. hedraiandra*, and *P. pachypleura* are here reported for the first time in the natural ecosystems of Portugal, whereas for *P. thermophila*, this is the first report from declining forests in Europe.

## 5. Conclusions

Overall, the results obtained have contributed to expanding scientific knowledge about the diversity of *Phytophthora* in Portuguese forest ecosystems. Portugal is a country characterized by extremely varied climatic and vegetation conditions, with a high human impact on land management. The coasts and surrounding areas are strongly related to the Mediterranean Sea and the Atlantic Ocean, while the internal part has continental influences typical of the Iberian Peninsula. To date, *Phytophthora* research in Portugal has been conducted chiefly in temperate and Mediterranean areas in the central and southern parts of the country, concentrating on cork oak, chestnut, and eucalyptus.

The results obtained in this study revealed the occurrence of *Phytophthora*-related diseases on many other tree and shrub species, contributing to expanding the knowledge about the impact of *Phytophthora* species on natural ecosystems. A total of 34 species and 3 hybrids of *Phytophthora* are now officially reported in Portugal.

## Figures and Tables

**Table 1:** Details of study sites and the samples collected.

Study Site	Climate Zone	Elevation (m a.s.l.)	Coordinates		Number of Samples	
			Latitude	Longitude	Rhizosphere	Necrotic Tissues
1	A	40	37.118918	−8.567076	Pl(5), Pp(2), Qc(2), Eg(1)	-
2	A	50	37.162606	−8.251300	Ces(3), Ph(1)	-
3	A	4	41.071134	−8.657429	Ce(1)	-
4	A	0	40.598795	−8.755363	Al(6), Ce(2)	Ce(2)
5	B	12	40.724190	−8.570960	Psp(6)	-
6	B	16	40.718277	−8.569993	Qr(3), Ssp(2)	Qr(1)
7	B	6	40.704444	−8.607099	Fa(2), Al(1)	-
8	B	5	40.695340	−8.633084	Eg(3), Fa(1)	-
9	B	18	40.551664	−8.575524	Eg(3)	-
10	B	437	41.554036	−8.375251	Qr(7), Qs(2)	-
11	B	400	38.783018	−9.416210	Cs(5), Ap(4), Qc(3), Rp(2), Eg(1)	Rp(21), Vm(2), Vt(4)
12	B	300	38.781366	−9.386651	Cs(7), Qr(6), Qs(2), Fm(2)	-
13	C	1056	40.490891	−7.520354	Bc(4)	-
14	C	500	40.612330	−7.519114	Qp(1)	-
15	C	1092	40.442888	−7.511881	Fe(2), Bc(1), Ps(1)	-
16	C	1107	40.541458	−7.454340	Cs(2), Qp(1)	-
17	C	1300	40.299541	−7.537996	Ld(2), Sa(1)	-
18	C	1450	40.328321	−7.587890	Bc(2), Ld(1)	-
19	C	1900	40.332200	−7.611709	Jc(2)	Jc(2)
20	C	900	40.327858	−7.677459	Cs(1)	-
21	C	680	40.383422	−7.700445	Cs(2)	-

In brackets the number of plants collected: *Acacia longifolia* (Al), *Acer pseudoplatanus* (Ap), *Betula celtiberica* (Bc), *Castanea sativa* (Cs), *Carpobrotus edulis* (Ce), *Ceratonia siliqua* (Ces), *Eucalyptus globulus* (Eg), *Ficus macrophylla* (Fm), *Fraxinus angustifolia* (Fa), *Fraxinus excelsior* (Fe), *Juniperus communis* (Jc), *Larix decidua* (Ld), *Pinus halepensis* (Ph), *Pistacia lentiscus* (Pl), *Pinus pinea* (Pp), *Pinus sylvestris* (Ps), *Populus* sp. (Psp), *Quercus coccifera* (Qc), *Quercus pyrenaica* (Qp), *Quercus robur* (Qr), *Quercus suber* (Qs), *Rhododendron ponticum* (Rp), *Sorbus aucuparia* (Sa), *Salix* sp. (Ssp), *Vinca major* (Vm), *Viburnum tinus* (Vt).

**Table 2:** Details of isolates included in the phylogenetic analyses. Ex-type cultures are given in bold and newly generated sequences are indicated in italics.

Species	ITS Clade	Collection No.	Host	ITS GenBank Accession No.
*Phytophthora cactorum*	1	**CBS231.30**	*Syringa vulgaris*	MG783385
*P. cactorum*	1	CBP168	*Castanea sativa*	*PQ571399*
*P. hedraiandra*	1	**CBS111725**	*Viburnum* sp.	MG865504
*P. hedraiandra*	1	CBP188	*Betula celtiberica*	*PQ571404*
*P. citricola*	2	**CBS221.88**	*Citrus sinensis*	MG865475
*P. citricola*	2	CBP150	*Acer pseudoplatanus*	*PQ571402*
*P. multivora*	2	**CBS124094**	*Eucalyptus marginata*	FJ237521
*P. multivora*	2	CBP154	*C. sativa*	*PQ571407*
*P. pachypleura*	2	**IMI502404**	*Aucuba japonica*	KC855330
*P. pachypleura*	2	CBP158	*C. sativa*	*PQ571408*
*P. plurivora*	2	**CBS124093**	*Fagus sylvatica*	MG865568
*P. plurivora*	2	CBP164	*C. sativa*	*PQ571409*
*P. ilicis*	3	**P3939**	*Ilex aquifolium*	MG865511
*P. pseudosyringae*	3	**CBS111772**	*Quercus robur*	MG865574
*P. pseudosyringae*	3	CBP195	*B. celtiberica*	*PQ571411*
*P. alticola*	4	**CBS141718**	*Eucalyptus grandis*	KX247599
*P. palmivora*	4	**CBS305.62**	*Areca catechu*	LC595875
*P. cocois*	5	**P19948**	*Cocos nucifera*	MG865478
*P. heveae*	5	**CBS296.29**	*Hevea brasiliensis*	MG865505
*P. amnicola*	6	**CBS131652**	water	JQ029956
*P. amnicola*	6	CBP134	*Rhododendron ponticum*	*PQ571396*
*P. asparagi*	6	**CBS132095**	*Lomandra sonderi*	EU301168
*P. asparagi*	6	CBP179	*Pistacia lentiscus*	*PQ571397*
*P. bilorbang*	6	**CBS161653**	*Rubus anglicandicans*	JQ256377
*P. bilorbang*	6	CBP140	*R. ponticum*	*PQ571398*
*P. chlamydospora*	6	**P6133**	*Prunus* sp.	MG865471
*P. chlamydospora*	6	CBP148	*A. pseudoplatanus*	*PQ571400*
*P. gonapodyides*	6	**P7050**	*Alnus* sp.	MG865501
*P. gonapodyides*	6	CBP186	*B. celtiberica*	*PQ571403*
*P. inundata*	6	**CBS216.85**	*Salix matsudana*	MG865516
*P. inundata*	6	CBP78	*Eucalyptus globulus*	*PQ571405*
*P. lacustris*	6	**IMI389725**	*S. matsundana*	JQ626605
*P. lacustris*	6	CBP162	*Populus* sp.	*PQ571406*
*P. rosacearum*	6	**CBS124696**	*Malus* sp.	EU925376
*P. rosacearum*	6	CBP116	*Acacia longifolia*	*PQ571412*
*P. thermophila*	6	**CBS127954**	*E. marginata*	EU301155
*P. thermophila*	6	CBP90	*Q. robur*	*PQ571414*
*P. cinnamomi*	7	**CBS144.22**	*Cinnamomum burmannii*	MG865473
*P. cinnamomi*	7	CBP185	*C. sativa*	*PQ571401*
*P. niederhauserii*	7	**CBS149824**	*Hedera helix*	MG865552
*P. pseudocryptogea*	8	**CBS139749**	*Isopogon buxifolius*	KP288376
*P. pseudocryptogea*	8	CBP166	*C. sativa*	*PQ571410*
*P. syringae*	8	**CBS110161**	*S. vulgaris*	MG865590
*P. syringae*	8	CBP220	*Q. robur*	*PQ571413*
*P. parsiana*	9	**IMI395329**	*Ficus carica*	MG865562
*P. polonica*	9	**CBS119650**	*Alnus glutinosa*	AB511828
*P. boehmeriae*	10	**CBS 291.29**	*Boehmeria nivea*	MG783382
*P. kernoviae*	10	**IMI393170**	*F. sylvatica*	AY940661
*P. lilii*	11	**CBS135746**	*Lilium longiflorum*	MG865523
*P. castanetorum*	12	**CBS142299**	*C. sativa*	MF036182
*P. quercina*	12	**CBS784.95**	*Q. robur*	MG865578
*Halophytophthora avicennae*	**-**	**CBS188.85**	*Avicennia marina*	HQ643147
*H. avicenniae*	**-**	CBP98	*E. globulus*	*PQ571415*
*Nothophytophthora caduca*	**-**	**CBS142350**	water	KY788401
*N. caduca*	**-**	CBP163	*Vinca major*	*PQ571416*

**Table 3:** Symptoms observed on each plant host and disease incidence/mortality rate estimated.

Plant Species	Symptoms Observed	Disease Incidence (%)	Mortality Rate (%)
*Acacia longifolia*	Root rot, bleeding cankers, canopy decline, sudden death	60–83	15–28
*Acer pseudoplatanus*	Root rot, exudates, bleeding cankers, chlorosis, stunted growth	nd	nd
*Betula celtiberica*	Root rot, bleeding cankers, canopy decline, sudden death	95	55
*Carpobrotus edulis*	Leaf necrosis, wilting	nd	nd
*Castanea sativa*	Root rot, bleeding cankers, chlorosis, canopy decline, sudden death	60–100	17–26
*Ceratonia siliqua*	Root rot, chlorosis, canopy decline	nd	nd
*Eucalyptus globulus*	Root rot, bleeding cankers, chlorosis, canopy decline, sudden death	80	20
*Ficus macrophylla*	Root rot, bleeding cankers, chlorosis, canopy decline, sudden death	nd	nd
*Fraxinus angustifolia*	Root and collar rot, canopy decline	nd	nd
*Fraxinus excelsior*	Root rot, canopy decline	nd	nd
*Juniperus communis*	Shoot blight, sudden death	nd	nd
*Larix decidua*	Root rot, chlorosis, canopy decline, sudden death	50	10
*Pistacia lentiscus*	Root rot, chlorosis, canopy decline, sudden death	76	31
*Pinus halepensis*	Root rot, sudden death	nd	nd
*Pinus pinea*	Root rot, sudden death	nd	nd
*Pinus sylvestris*	Root rot, canopy decline	nd	nd
*Populus* sp.	Root rot, chlorosis, canopy decline, sudden death	70	12
*Quercus coccifera*	Root rot, canopy decline	60	17
*Quercus pyrenaica*	Root rot, canopy decline	nd	nd
*Quercus robur*	Root rot, bleeding cankers, chlorosis, canopy decline, sudden death	67	11
*Quercus suber*	Root rot, bleeding cankers, chlorosis, canopy decline, sudden death	nd	nd
*Rhododendron ponticum*	Leaf necrosis, wilting, shoot blight, root rot, sudden death	100	16
*Salix* sp.	Root rot, chlorosis, canopy decline, sudden death	75	22
*Sorbus aucuparia*	Root rot, canopy decline	nd	nd
*Vinca major*	Leaf necrosis, wilting	nd	nd
*Viburnum tinus*	Leaf necrosis, wilting	nd	nd

nd = not determined.

**Table 4:** Number of isolates obtained from the monitored plants in the investigated sites.

Species	ITS Clade	Plant Species *	Total Number of Isolates	Sites
*Halophytophthora* *avicenniae*	-	Eg(2)	2	8
*Nothophytophthora* *caduca*	-	Vm(2)	2	11
*Phytophthora amnicola*	6	Rp(5), Qr(1)	6	11, 12
*P. asparagi*	6	Pl(3)	3	1
*P. bilorbang*	6	Rp(4)	4	11
*P. cactorum*	1	Qr(5), Qs(1) Cs(1), Jc(1)	8	10, 12, 19
*P. chlamydospora*	6	Qs(1), Ap(1)	2	10,11
*P. cinnamomi*	7	Eg(7), Qr(6), Qc(5), Bc(4), Qs(2), Al(2), Cs(3), Ce(3), Rp(1), Ces(1), Pl (1), Pp(1)	36	1–4, 6, 10–13, 16, 20, 21
*P. citricola*	2	Ap(3)	3	11
*P. gonapodyides*	6	Bc(2), Al(1), Qr(1)	4	4, 10, 18
*P. hedraiandra*	1	Rp(3), Vm(2), Bc(1)	6	11, 18
*P. inundata*	6	Eg(2), Qr(2)	4	6, 8
*P. lacustris*	6	Ssp(2), Psp(4), Qr(2)	8	5, 6, 10
*P. multivora*	2	Rp(13), Cs(3), Fm(2), Fa(1), Al(1)	20	4, 7, 11, 12
*P. pachypleura*	2	Cs(2)	2	12
*P. plurivora*	2	Qr(3), Cs(2), Fe(1), Bc(1), Qp(1), Eg(1)	9	10, 12, 14–16
*P. pseudocryptogea*	8	Al(2), Pl(3), Ph(1), Cs(2)	7	1, 2, 4, 11, 12
*P. pseudosyringae*	3	Jc(2), Bc(1), Sa(1), Ld(1)	5	13, 17, 19
*P. rosacearum*	6	Al(3)	3	4, 7
*P. syringae*	8	Qr(1)	1	6
*P. thermophila*	6	Pp(2), Rp(1), Qr(1), Qs(1)	5	1, 10, 11

* In brackets, the number of *Phytophthora* isolates on: *Acacia longifolia* (Al), *Acer pseudoplatanus* (Ap), *Betula celtiberica* (Bc), *Castanea sativa* (Cs), *Carpobrotus edulis* (Ce), *Ceratonia siliqua* (Ces), *Eucalyptus globulus* (Eg), *Ficus macrophylla* (Fm), *Fraxinus angustifolia* (Fa), *Fraxinus excelsior* (Fe), *Juniperus communis* (Jc), *Larix decidua* (Ld), *Pinus halepensis* (Ph), *Pistacia lentiscus* (Pl), *Pinus pinea* (Pp), *Pinus sylvestris* (Ps), *Populus* sp. (Psp), *Quercus coccifera* (Qc), *Quercus pyrenaica* (Qp), *Quercus robur* (Qr), *Quercus suber* (Qs), *Rhododendron ponticum* (Rp), *Salix* sp. (Ssp), *Sorbus aucuparia* (Sa), *Vinca major* (Vm), and *Viburnum tinus* (Vt).

**Table 5:** *Phytophthora* species reported in natural and semi-natural ecosystems in Portugal.

Species	Host	References
*P. alticola*	*Eucalyptus globulus*	[29]
*P. amnicola*	*Alnus glutinosa*, *Castanea sativa*, *Fagus sylvatica*, *Rhododendron ponticum*, *Quercus robur*	[27,38]; this study
*P. asparagi*	*A. glutinosa*, *Pistacia lentiscus*	[27]; this study
*P. bilorbang*	*R. ponticum*, water	[27,38]; this study
*P. cactorum*	*A. glutinosa*, *Castanea sativa*, *Quercus robur*, *Q. suber*, *Juniperus communis*	[27]; this study
*P. cambivora*	*Acer pseudoplatanus*, *Betula celtiberica*, *C. sativa*, *F. sylvatica*, *Salix caprea*, *Quercus ilex*, *Quercus pyrenaica*, water	[38,47,48,49]
*P. castanetorum*	*C. sativa*	[28,38]
*P. chlamydospora*	*A. pseudoplatanus*, *A. glutinosa*, *C. sativa*, *F. sylvatica*, *Q. suber*	[27,38]; this study
*P. cinnamomi*	*Abies alba*, *Acacia longifolia*, *A. glutinosa*, *Arbutus unedo*, *B. celtiberica*, *Calluna vulgaris*, *Carpobrotus edulis*, *C. sativa*, *Ceratonia siliqua*, *Cistus crispus*, *C. ladanifer*, *C. populifolius*, *C. salvifolius*, *E. globulus*, *F. sylvatica*, *Genista triacanthos*, *Phyllirea latifolia*, *Pinus pinaster*, *P. pinea*, *P. lentiscus*, *Quercus coccifera*, *Q. ilex*, *Q. pyrenaica*, *Q. robur*, *Quercus rubra*, *Q. suber*, *R. ponticum*, *Ulex* spp.	[27,29,30,31,32,34,38,50,51]; this study
*P. citricola*	*A. pseudoplatanus*	This study
*P. citrophthora*	water	[38,52]
*P. condilina*	water	[26]
*P. gonapodyides*	*A. longifolia*, *A. glutinosa*, *B. celtiberica*, *Q. robur*, water	[26,27,38]; this study
*P. hedraiandra*	*B. celtiberica*, *R. ponticum*, *Vinca major*	This study
*P. hibernalis*	*E. globulus*	[30]
*P. honggalleglyana*	water	[38]
*P. inundata*	*E. globulus*, *Q. robur*, water	[26,38]; this study
*P. kelmanii*	water	[38]
*P. lacustris*	*A. glutinosa*, *Populus* sp., *Q. robur*, *Salix* sp., water	[27,37,38]; this study
*P. multivora*	*Acacia dealbata*, *A. longifolia*, *A. glutinosa*, *A. pseudoplatanus*, *C. sativa*, *E. globulus*, *Fraxinus angustifolia*, *Q. rubra*, *R. ponticum*	[27,30,38]; this study
*P. niederhauserii*	*E. globulus*	[30]
*P. pachypleura*	*C. sativa*	This study
*P. plurivora*	*A. glutinosa*, *A. pseudoplatanus*, *B. celtiberica*, *C. sativa*, *F. sylvatica*, *Fraxinus excelsior*, *Prunus lusitanica*, *Q. pyrenaica*, *Q. robur*	[27,38]; this study
*P. polonica*	*A. glutinosa*	[27]
*P. pseudocitrophthora*	water	[38,52]
*P. pseudocryptogea*	*A. dealbata*, *A. longifolia*, *A. glutinosa*, *C. sativa*, *P. lentiscus*, *Pinus halepensis*, *P. pinea*, *Q. suber*, water	[26,27,52]; this study
*P. pseudosyringae*	*A. pseudoplatanus*, *B. celtiberica*, *J. communis*, *Larix decidua*, *Q. pyrenaica*, *Prunus avium*, *Sorbus aucuparia*	[38,49]; this study
*P. psychrophila*	*Q. ilex*	[38,49]
*P. quercina*	*A. unedo*, *C. sativa*, *Q. ilex*, *Q. pyrenaica*, *Q. robur*	[28,38]
*P. ramorum*	*Viburnum* sp.; water	[38,53]
*P. rosacearum*	*A. longifolia*, *A. glutinosa*	[27]; this study
*P. syringae*	*A. unedo; Q. robur*	[38]; this study
*P. thermophila*	*P. pinea*, *Q. robur*, *Q. suber*, *R. ponticum;* water	[38]; this study
*P. uliginosa*	*Q. suber*	[38]
*P. × alni*	*A. glutinosa*	[37,38]
*P. × lusitanica*	water	[38,52]
*P. × stagnum*	water	[38]

## Data Availability

The original contributions presented in the study are included in the article, further inquiries can be directed to the corresponding author.
